# Optical and magneto-optical behavior of Cerium Yttrium Iron Garnet thin films at wavelengths of 200–1770 nm

**DOI:** 10.1038/srep23640

**Published:** 2016-03-30

**Authors:** Mehmet C. Onbasli, Lukáš Beran, Martin Zahradník, Miroslav Kučera, Roman Antoš, Jan Mistrík, Gerald F. Dionne, Martin Veis, Caroline A. Ross

**Affiliations:** 1 Department of Materials Science and Engineering, Massachusetts Institute of Technology, 77 Massachusetts Avenue, MIT Cambridge, MA 02139, USA; 2 Charles University of Prague, Faculty of Mathematics and Physics, Ke Karlovu 3, 12116 Prague 2, Czech Republic; 3 University of Pardubice, Faculty of Chemical Technology, Institute of Applied Physics and Mathematics, Studentska 95, 53210 Pardubice, Czech Republic

## Abstract

Magneto-optical cerium-substituted yttrium iron garnet (Ce:YIG) thin films display Faraday and Kerr rotation (rotation of light polarisation upon transmission and reflection, respectively) as well as a nonreciprocal phase shift due to their non-zero off-diagonal permittivity tensor elements, and also possess low optical absorption in the near-infrared. These properties make Ce:YIG useful in providing nonreciprocal light propagation in integrated photonic circuits, which is essential for accomplishing energy-efficient photonic computation and data transport architectures. In this study, 80 nm-thick Ce:YIG films were grown on Gadolinium Gallium Garnet substrates with (100), (110) and (111) orientations using pulsed laser deposition. The films had bulk-like structural and magnetic quality. Faraday and Kerr spectroscopies along with spectroscopic ellipsometry were used to deduce the complete permittivity tensor of the films in the ultraviolet, visible and near-infrared spectral region, and the magneto-optical figure of merit as a function of wavelength was determined. The samples showed the highest IR Faraday rotation reported for thin films of Ce:YIG, which indicates the importance of this material in development of nonreciprocal photonic devices.

## Introduction

Materials and photonic device engineering over the past decade have made major strides towards assembling a monolithically-integrated optical signal processor by developing integrated Group IV lasers^1^, photodetectors^2^, filters and resonators^3^, and modulators^4^. Developing these systems using Group IV materials enables integration on an inexpensive silicon platform using well-established and compatible silicon device processing techniques. One of the major requirements in completing a fully-integrated optical signal processor is integrating an optical isolator on-chip with the other photonic components in order to establish signal stability^5^ and to avoid back-reflection of optical and near-infrared pulses from other components of the circuit into the gain region of the laser. Optical isolators include a magneto-optical (MO) material layer which breaks time-reversal symmetry and enables nonreciprocal photonic transmission (i.e. one way transmission of light). The most commonly used MO layers for optical isolators are cerium- or bismuth-substituted yttrium iron garnet (Ce:YIG or Bi:YIG, (Ce or Bi)_x_Y_3-x_Fe_5_O_12_) which have high MO figure of merit (°dB^−1^), which is defined as Faraday rotation, in units of °cm^−1^, divided by optical loss, in dB cm^−1^. These materials have high optical transmission as well as strong Faraday rotation in the IR^5^,^6^.

A major roadblock for completing a fully-integrated optical signal processor has been the processing of MO garnet films which is necessary to integrate optical isolators onto photonic circuits based on Si or other substrates. Garnet films have large lattice parameters and large thermal expansion mismatch with typical photonic substrates such as Si, GaAs, or InP^6^. Good quality films have been made by controlling the deposition and annealing processes and by using seed layers^6^,^7^,^8^,^9^,^10^,^11^,^12^,^13^,^14^,^15^,^16^,^17^,^18^,^19^,^20^,^21^,^22^,^23^, enabling demonstrations of nonreciprocal photonic devices such as isolators and modulators^24^,^25^,^26^,^27^,^28^,^29^,^30^,^31^,^32^.

To guide the design of photonic devices operating at different wavelengths, including atomic clocks, inertial sensors, optical phased arrays, or quantum computation systems in addition to optical isolators, modulators and and circulators, and to enable comparisons between materials and processing techniques, it is important to determine the best achievable optical and magnetooptical properties across a wide spectral range, i.e. to determine the full permittivity tensor as a function of wavelength, especially in the visible and near-infrared and for films with well characterized bulk-like structure and magnetic properties. Here, we present the complete optical characterization of thin CeYIG films Ce:YIG (Ce_1_Y_2_Fe_5_O_12_) films grown on gadolinium gallium garnet (Gd_3_Ga_5_O_12_, GGG) substrates over visible and near infrared bands. Among commercially available substrates, GGG (a = 12.376 Å) was preferred because it has a lattice parameter close to that of Ce:YIG (a = 12.57 Å)^33^.

Most of the previous reports of Ce:YIG films include figure of merit measurement results only for a single wavelength or for a few sample wavelengths within a photonic band^5^,^6^,^7^,^8^,^16^,^19^,^21^,^22^,^23^,^24^,^25^,^26^,^27^,^28^,^29^. Gomi *et al.*^34^ presented wavelength-dependent Faraday rotation and optical absorption for 1 eV to 2.6 eV photon energy (λ = 480–1240 nm) but this publication did not include data for the near-infrared communication wavelengths. Despite the interest in using garnet films for nonreciprocal photonic devices, to the best of our knowledge, there is no comprehensive report of the optical properties and magneto-optical figure of merit of Ce:YIG as a function of wavelength. The knowledge of these variables, which are captured in the diagonal and off-diagonal elements of the permittivity tensor, is necessary to design and optimize devices such as integrated magneto-optical isolators.

In the following paragraphs, first, the growth of the films is described. Next; structural, magnetic hysteresis and magnetic anisotropy of Ce:YIG films on GGG (100), (110) and (111) orientations are presented. Finally, the spectral dependence of the complete permittivity tensor of Ce:YIG is presented for the energy range 0.7–6 eV. The permittivity tensor of a magnetic material in a magnetic field perpendicular to its film surface can be written in the form (with restriction to linear magneto-optical effects)^35^


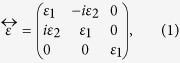


where ε_1_ and ε_2_ are complex numbers with real and imaginary parts describing the optical and magneto-optical response of the material. This tensor form can be used for a theoretical description of both Faraday and polar Kerr magneto-optical effects since the magnetization is oriented in the same direction.

### Ce:YIG film growth on GGG substrates

Ce:YIG films were grown on GGG (100), (110) and (111) double-side polished substrates by pulsed laser deposition (PLD) using a 248 nm wavelength KrF excimer laser (Coherent, COMPex Pro 205). Ce:YIG oxide targets were fabricated as described previously^7^. Deposition parameters, base pressure, oxygen pressure during growth, deposition and cooling rates are presented in the methods section. Ce:YIG films of 80 nm thicknesses (measured by X-ray reflectivity (XRR) and validated by spectroscopic ellipsometry) were deposited with a laser pulse rate of 10 Hz at 615 °C substrate temperature. Ce:YIG was deposited simultaneously on all three substrate orientations. Peak-to-valley surface roughness of the Ce:YIG films was within 1 nm. Control of oxygen pressure and substrate temperature can preserve the stoichiometry and prevent the formation of secondary phases such as ceria, as previously discussed^6^. After the deposition process, no further thermal annealing step was carried out.

## Results and Discussion

### Structural and magnetic characterization of Ce:YIG films

The crystalline phase content of the films was characterized using XRD and a silicon zero-background holder. Ce:YIG and GGG lattice parameters are close and hence the Ce:YIG and GGG peaks were separated by 0.2–0.5°. The thickness of the films was determined to be 80 nm by XRR measurements. The *ω*-2*θ* plots, shown in Supplementary Fig. S1(a), were measured for an 80 nm thickness Ce:YIG on GGG (100) and a GGG (100) substrate with no film. The plot shows clearly the (400) peak originating from the film; other peaks are from the substrate. Supplementary Fig. S1(b,c) show corresponding data from films on (110) and (111) substrates, respectively. The patterns in Supplementary Fig. S1(c) are similar to the garnet film pattern on GGG (111) reported previously^36^. There are no detectable secondary phases.

**Figure 1:**
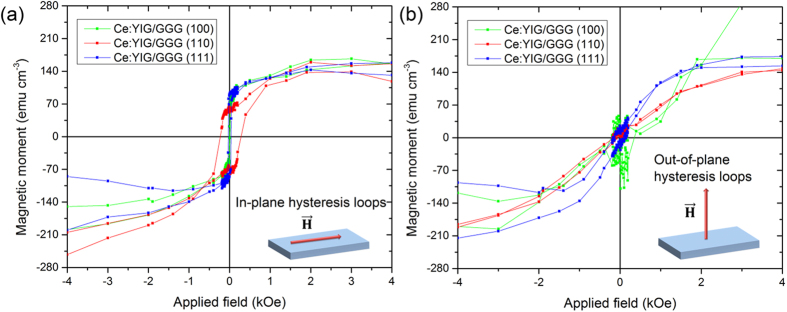
Room temperature magnetic hysteresis loops of Ce:YIG (80 nm) films on GGG (100), GGG (110), and on GGG (111) substrates with magnetic field applied (**a**) in plane and (**b**) perpendicular to the film plane. All films have in-plane magnetic easy axis.

Magnetic properties of Ce:YIG films were measured with magnetic fields applied parallel to the film plane (in-plane configuration, IP) or perpendicular to the film plane (OP) at room temperature (RT) using a vibrating sample magnetometer (VSM). IP magnetization hysteresis loops for the three films are shown in Fig. 1(a) and OP loops are shown in Fig. 1(b). The saturation magnetization was M_s_ = 150 ± 15 emu cm^−3^, consistent with previous reports^6^,^22^. Noise and drift in the measurements are due to the high paramagnetic background signal of GGG, especially during high field measurements (|H_applied_| > 6000 Oe). This does not, however, affect the magneto-optical measurements.

All of the films had their easy axes in-plane regardless of the substrate type. From Fig. 1(b), the hard axis saturation field was lowest at ~2 kOe for the (111) film, ~5 kOe for the (110) film, and ~8 kOe for the (100) film. Shape anisotropy (K_s_ = 4πM_s_), magnetocrystalline and magnetoelastic anisotropy contribute to the total anisotropy of the films. The magnetoelastic anisotropy arises from the lattice distortion (c/a ratio ~1.015 for the (100) sample, with the film in in-plane compression) due to epitaxial growth. The differences in hard-axis saturation field originate from the angular dependence of magnetocrystalline and magnetoelastic anisotropy. The anisotropy contributions in Ce:YIG films are described in greater detail elsewhere^33^. A more detailed description of the substrate orientation and angular dependence of the anisotropy of IP magnetic hysteresis loops are presented in the Supplementary Fig. S2.

### Wavelength and substrate orientation-dependent optical characterization of Ce:YIG films

**Figure 2:**
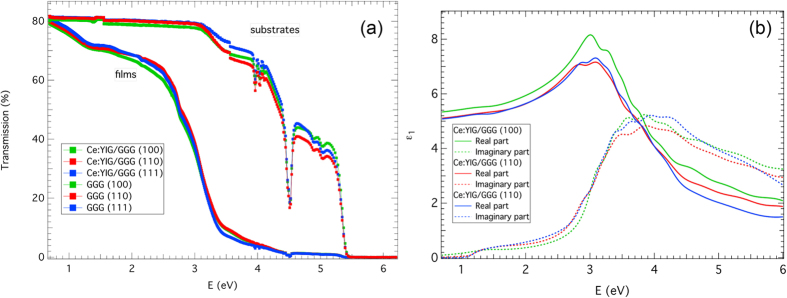
(**a**) Room temperature optical and near-infrared transmission of Ce:YIG (80 nm) films on GGG substrates. Absorption peaks of the GGG substrate near 4.504 eV, 4.035 eV, 3.972 eV originate from the intrinsic electronic transitions of Gd^3+^ ions (from the ^8^S ground state to ^6^I, ^6^P_5/2_ and ^6^P_7/2_, respectively). Absorption near IV (1.4 eV) belongs to the film. (**b**) Spectral dependence of diagonal permittivity elements, ε_1_, of three Ce:YIG samples on GGG substrates.

First, the electronic band gap and optical transmission of the films and uncoated substrates are shown in Fig. 2. GGG has a band edge at λ_g_ = 232 nm (E_g,GGG_ = 5.344 eV) for all substrate orientations. GGG also has a band tail that extends from 5.344 eV to 1.653 eV, with sharp absorption peaks at E = 4.504 eV, 4.035 eV, 3.972 eV due to intrinsic electronic transitions in the GGG lattice. 4.504 eV (36324 cm^−1^), 4.035 eV (32514.5 cm^−1^) and 3.972 eV (31928.5 cm^−1^) transitions belong to the 4 f transitions of Gd^3+^ ions from the ^8^S ground state to ^6^I, ^6^P_5/2_ and ^6^P_7/2_, respectively^37^,^38^. These energy transitions originate from spin-orbit and crystal field splitting. The transmission spectra for 80 nm-thick films shown in Fig. 2(a) indicates that Ce:YIG has an indirect band-gap similar to GGG, but with a stronger absorption tail reaching into the visible. There is also a weak but non-zero refractive index difference between the substrates with different orientations.

The spectral dependence of ε_1_ for all samples is shown in Fig. 2(b). This dependence is similar to that of YIG single crystals and thin films^39^,^40^,^41^,^42^. The main difference is in the energy region between 1 and 2 eV, where the absorption peak centered near 1.4 eV is visible in the spectra of the imaginary part of ε_1_. This absorption arises from the intra-ionic electrical dipole transitions of the Ce^3+^ ions and is also observable in the transmission spectra in Fig. 2(a). The fitted film thicknesses were in a good agreement with the results of XRR. The slight difference (up to 5 nm) can be attributed to the surface roughness, which has a different influence on X-ray and optical measurements.

**Figure 3:**
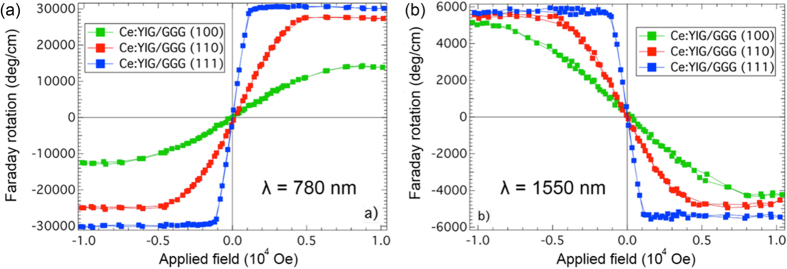
Room temperature Faraday rotation hysteresis loops of Ce:YIG films at (**a**) λ = 780 nm, and (**b**) λ = 1550 nm.

Complex Faraday magneto-optical spectra and hysteresis loops were obtained from a spectrometer based on the azimuth modulation technique (rotating polarizer). Faraday loops at wavelengths 780 nm and 1550 nm are displayed in Fig. 3. The loops are consistent with VSM measurements shown in Fig. 1(b), with anisotropy fields of around H_K_ = 8 kOe, 5 kOe and 2 kOe for (100), (110) and (111) GGG substrates respectively. The anisotropy field for the (111) sample is close to that expected from shape anisotropy (compare 1780 Oe for YIG^43^), but higher anisotropy fields for films on (100) and (110) GGG substrates suggest the influence of magnetocrystalline (for (110)) or magnetoelastic anisotropies for those orientations.

The Faraday rotation was substantial, especially considering the small film thickness. For example, for the (111) film the FR reached 30,000° cm^−1^ at 780 nm and −5800° cm^−1^ at 1550 nm. This exceeds values reported for bulk and thin film Ce-substitued YIG^5^,^6^,^7^,^8^,^10^,^12^,^13^,^15^,^16^,^21^,^22^,^23^,^25^,^26^,^27^,^28^,^29^,^30^,^32^,^33^,^34^,^44^,^45^,^46^,^47^,^48^,^49^. For comparison Shintaku *et al.* reported FR = −3300° cm^−1^ to −3800° cm^−1^ at 1550 nm for sputtered epitaxial films of Ce:YIG with a composition of x = 1^23^ and Gomi *et al.*^47^ gave a FR of 13,000° cm^−1^ at 1150 nm for x = 1 in a sputtered 1 μm thick film, though the absorption was not measured.

**Figure 4:**
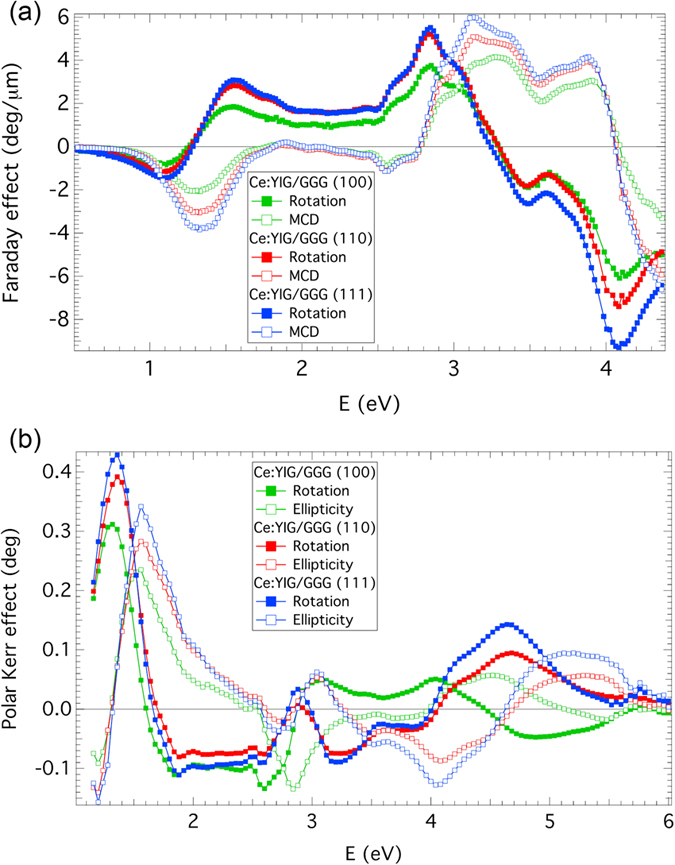
(**a**) Room temperature Faraday rotation and MCD spectra of 80 nm thick Ce:YIG films on GGG (100), (110) and (111) substrates. (**b**) Room temperature polar Kerr rotation and ellipticity spectra of 80 nm thick Ce:YIG films on GGG (100), (110) and (111) substrates.

The Faraday rotation and magnetic circular dichroism (MCD) are shown in Fig. 4. A similar spectral dependence was reported on micrometer-thick Ce:YIG layers with high Ce^3+^ content^46^,^47^,^48^. The spectra exhibit clearly visible spectroscopic structure situated near 1.4 eV similarly to the optical transmission and ellipsometric results. This comes from the 4f–5d transition in Ce^3+^ ions^49^,^50^. The Ce^3+^(4f)–Fe^3+^(tetrahedral) electric dipole transition between 1–2 eV is considered to be the origin of the enhancement in the near-infrared (i.e. λ = 1550 nm) magneto-optical effect in Ce:YIG compared to YIG^5^,^34^. Other spectroscopic structures in the Faraday spectra are located near 3.1 and 3.9 eV originating from Fe^3+^ crystal-field transitions already well described in the literature^51^. The amplitudes of the Faraday effect in Fig. 4 clearly show (consistently with hysteresis loops measurements) the Ce:YIG film on (111) GGG substrate to be of the highest quality among the three samples.

Magneto-optical Kerr spectroscopy was performed using a general magneto-optical ellipsometer with rotating analyser in a polar configuration at nearly normal incidence at room temperature. The magnetic field of 10 kOe was sufficient to magnetically saturate all the samples. Experimental polar Kerr spectra of the samples are displayed in Fig. 4(b). The spectra are dominated by strong magneto-optical response in the energy region below 2 eV. At this energy range the penetration depth in Ce:YIG exceeds the layer thickness, which results in propagation of light across the film and a double-Faraday effect contribution to the polar Kerr spectra. Although the Ce:YIG layers grown on (110) and (111) GGG substrates exhibit similar spectral behaviour, the Ce:YIG layer grown on (100) GGG substrate shows notable differences above 3 eV. At this spectral region the thickness exceeds the penetration depth of the material. Therefore, this difference, which is not visible in Faraday spectra in Fig. 4, should be related to the surface of the sample. This points to a damaged non-magnetic surface layer, which was probably formed when the sample was bonded for mechanical roughening of the backside of the substrate, which was required to carry out the MOKE measurements. AFM measurements confirmed a notable increase of the surface roughness with a peak to valley height of 18 nm. Effects of magnetically disordered surface layers have been reported in Bi:YIG thin polycrystalline films^52^ and YIG nanoparticles^53^.

**Figure 5:**
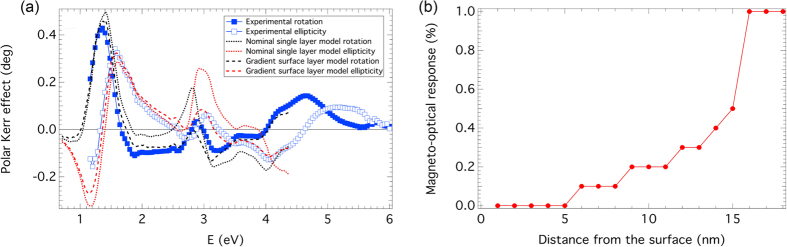
(**a**) Room temperature polar Kerr rotation and ellipticity spectra of an 80 nm thick Ce:YIG film on (111) GGG substrate compared with theoretical models. (**b**) The profile of the magneto-optical response of the surface layer used to model the polar Kerr data.

A 4 × 4 matrix formalism for description of light propagation in anisotropic multilayers^35^ was employed to derive the off-diagonal elements of the permittivity tensor, ε_2_, of the Ce:YIG films from the Faraday spectra. To validate the correctness of this derivation, polar Kerr effect spectra were calculated using the derived permittivity tensor. The comparison of the experimental data with theoretically calculated Kerr spectra for a Ce:YIG layer on (111) GGG substrate is displayed in Fig. 5. The nominal model structure of an 80 nm thick Ce:YIG layer on an infinite (111) GGG substrate described the experimental data reasonably in the IR region, however a notable difference was observed above 3 eV. Since in the spectral region above 3 eV, the polar Kerr effect carries information about the surface, a revised model structure considering a 15 nm thick surface layer, with gradually increasing magneto-optical response from 0 to 100% of Ce:YIG, and a 65 nm homogeneous Ce:YIG layer was considered to calculate the theoretical spectra of the polar Kerr effect. The result is shown in Fig. 5(a) and the profile of the magneto-optical response across the surface layer is shown in Fig. 5(b). An excellent agreement between the calculation and experimental data was achieved for the revised model structure. Similar results were obtained for the other samples, however the thickness of surface layer was estimated to be 35 and 15 nm for Ce:YIG layers on (100) and (110) GGG substrates. This is consistent with the lower amplitude of Faraday rotation in saturation in the (100) sample. These surface layer thicknesses agree well with AFM peak to valley heights resulting from the film damage during bonding.

**Figure 6:**
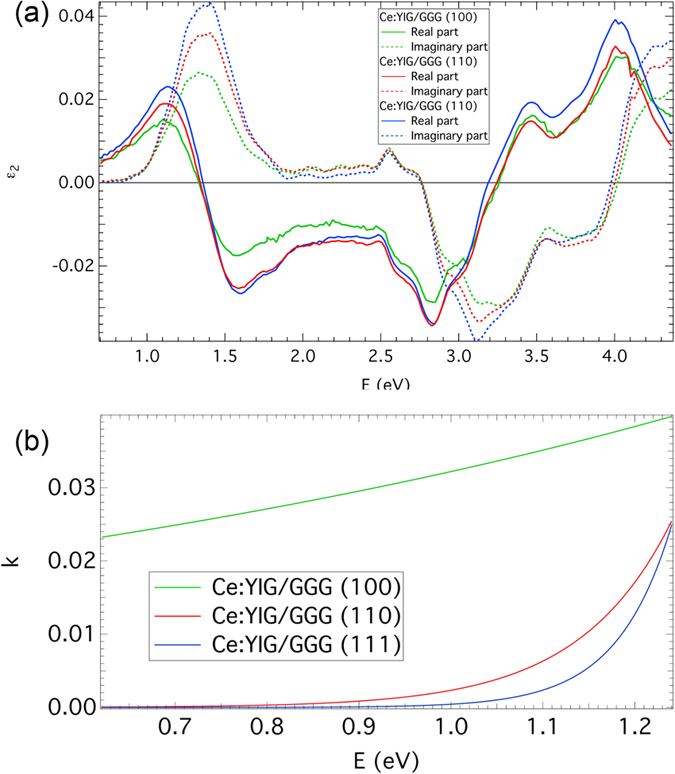
(**a**) Spectral dependence of off-diagonal permittivity elements, ε_2_, of three Ce:YIG samples on GGG substrates. (**b**) Spectral dependence of extinction coefficient of Ce:YIG films on GGG substrates.

Spectrally dependent off-diagonal permittivity elements, ε_2_, derived from Faraday spectra are displayed in Fig. 6(a). Since the Faraday rotation spectra were measured on as-deposited samples (without any back-surface roughening) and back-calculated polar Kerr spectra agree well with experimental results if the surface layer model structure was considered, the spectra of ε_2_ shown in Fig. 6(a) represent data from Ce:YIG without any non-magnetic phase or surface layer. The magneto-optical response in Ce:YIG is dominated by one spectroscopic structure near 1.4 eV and multiple structures above 2.6 eV. The low energy response with a paramagnetic shape is related to an intra-ionic electrical dipole transition of Ce^3+^ as already mentioned above. On the other hand at energies above 3 eV the magneto-optical properties of Ce:YIG are mainly driven by charge transfer transitions involving Fe^3+^ ions^51^.

To evaluate the performance of Ce:YIG in a nonreciprocal photonic device, knowledge of the exact value of absorption is necessary to calculate the figure of merit. At the main wavelength of interest, λ = 1550 nm (E = 0.8 eV), the absorption coefficient of the samples was very small. At such low values the fitting of pure ellipsometric experimental data is not effective and other complementary experiments are necessary^54^ to obtain reasonably accurate results. Therefore, additional transmission experiments in IR region using a highly precise Thermo Nicolet Nexus FTIR spectrometer were carried out on all samples. The IR transmission data were fitted using the results of spectroscopic ellipsometry. The refractive index was taken from ellipsometric experiments as input for the fitting procedure, as well as the thickness of the Ce:YIG layer. The optical dependence of the extinction coefficient, k, was then parameterized using Cauchy relations.

The resulting spectral dependences of k for all samples are shown in Fig. 6(b) and spectral dependences of ε_1_ are included in Fig. 2(b). The Ce:YIG film on GGG (100) shows a notable difference in the spectral dependence of extinction coefficient compared to films on GGG (110) and (111) substrates. The optical transmission of the Ce:YIG film on GGG does not depend only on the absorption coefficient of the film and substrate. Since it is an example of a thin layer between two media, multiple reflections in the Ce:YIG film considerably influence the total optical transmission of the sample. These reflections are driven by the ratio of refractive indices of Ce:YIG and GGG. From Fig. 2(a) and our ellipsometric measurements we have found that the optical properties of GGG slightly differ according to the GGG orientation. Moreover, the optical properties of GGG with the same crystallographic orientation differ for different substrate suppliers, which might be the consequence of different polishing methods. It is therefore possible that the actual optical properties in the IR region of the (100) GGG substrate with Ce:YIG film slightly differ from the optical properties of the bare substrate, which were used for theoretical modelling. This may have caused the higher absorption in the case of Ce:YIG on (100) GGG, which would otherwise be attributed to the optical effects at Ce:YIG layer/GGG substrate interface. To obtain information about such low absorption coefficients, other experimental techniques, such as photothermal deflection spectroscopy^55^, may give values with higher accuracy than standard spectrophotometric experimental techniques.

**Figure 7:**
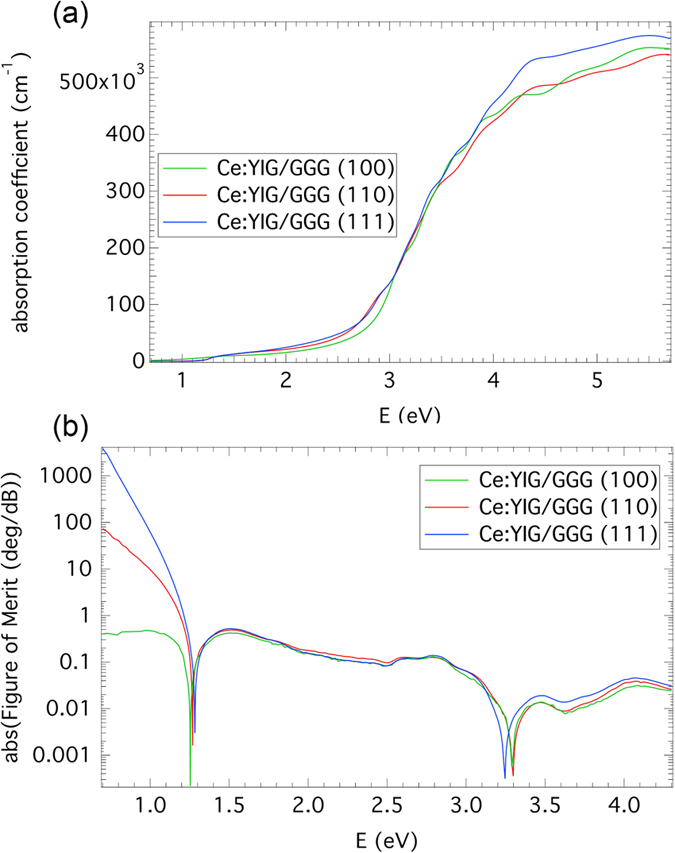
(**a**) Spectral dependence of absorption coefficient of Ce:YIG films on GGG substrates. (**b**) Spectral dependence of Figure of Merit of Ce:YIG films on GGG substrates.

The spectral dependences of the absorption coefficient and the magneto-optical figure of merit were calculated and are displayed in Fig. 7(a,b). The spectra exhibit a low absorption coefficient in the IR region leading to high values of the figure of merit at communication wavelengths around 1550 nm. Figures of merit were 31 and 943° dB^−1^ at E = 0.8 eV for Ce:YIG layers on (110) and (111) GGG substrates respectively. The one order difference in these values can be explained with the help of Figs 6(b) and 7(b). A visible shift of approx. 0.1 eV in the absorption to the higher energy side can be observed for the Ce:YIG film on (111) GGG. Since the wavelength of 1550 nm is in the vicinity of the absorption edge where the absorption is changing rapidly with the energy, even a small shift of this edge can result in a noticeable change in the IR absorption. Such change may be caused by the influence of the substrate orientation on the intra-ionic electrical dipole transitions of the Ce^3+^ or a slight variation in Ce^3+^ composition between the samples. The results can be compared to a previous study indicating that single-crystalline CeYIG thin films have 340° dB^−1^ (TE mode) and 540° dB^−1^ (TM mode) figure of merit for Ce:YIG on Gd_3_Sc_2_Ga_3_O_12_ substrates using a slab waveguide loss measurement technique which contains contributions from the substrate^23^. From Fig. 7 it also follows that for the same deposition conditions the growth of Ce:YIG on GGG substrates leads to the best quality when the (111) substrate orientation is used. It is important to note that the values of figure of merit were calculated considering the total thickness of 80 nm. Table 1 shows a comparison of MO figure of merit for Ce:YIG films and bulk crystals, magnetic ion-doped perovskites and Group III-V films demonstrated previously^5^,^7^,^16^,^23^,^25^,^44^,^45^,^56^,^57^,^58^,^59^,^60^.

## Conclusion

Epitaxial films of Ce:YIG (Ce_1_Y_2_Fe_5_O_12_) 80 nm thick were grown on (100), (110) and (111) GGG substrates. The films showed no evidence of non-garnet phases and a magnetization close to bulk. A complete optical and magneto-optical characterization in the spectral energy range from 0.7 to 6 eV was performed for different substrate orientations. The spectral dependence of the complete permittivity tensor of Ce:YIG was deduced from experimental data using theoretical modelling. The knowledge of this tensor is crucial for the design of novel devices for integrated photonics for communications as well as a host of other applications in the visible and near-IR.

Both Faraday and polar Kerr spectroscopies showed strong magneto-optical response at low energies where intra-ionic transitions of Ce^3+^ play a role, while at energies above 3 eV charge transfer transitions of Fe^3+^ ions are involved similarly to pure YIG. The values of Faraday rotation of −5800°/cm at 1550 nm wavelength are exceptionally high and exceed reported bulk values.

The high Faraday rotation and the low absorption in the IR region resulted in high values of figure of merit for parts of the spectral range, indicating the high quality of the CeYIG films, especially when the (111) GGG substrate was used, and confirms their suitability for applications in magneto-optical isolators and other devices, particularly around 1 eV and 1.6 eV. These results provide guidelines for the utility of Ce:YIG films in integrated photonic devices.

## Methods

### Ce:YIG film growth on GGG substrates

GGG (100), (110) and (111) double-side polished substrates (supplier: MTI Crystals) were cleaned using sonication in acetone and isopropanol for 10 minutes each. Ce:YIG films were grown by pulsed laser deposition (PLD) using a 248 nm wavelength KrF excimer laser (Coherent, COMPex Pro 205). Ce:YIG oxide targets were fabricated as described previously^7^. During PLD, the base pressure and oxygen pressure used for Ce:YIG growth were 5 × 10^−6^ Torr and 5 mTorr, respectively. Ce:YIG films of 80 nm thicknesses (measured by X-ray reflectivity (XRR) and validated by spectroscopic ellipsometry) were deposited with a laser pulse rate of 10 Hz at 615 °C substrate temperature, with a deposition rate of 18 Å min^−1^. Ce:YIG was deposited simultaneously on all three substrate orientations. The cooling rate of the chamber after deposition was 10 °C min^−1^. Peak-to-valley surface roughness of Ce:YIG films was within 1 nm. Control of oxygen pressure and substrate temperature can preserve the stoichiometry and prevent the formation of secondary phases such as ceria, as previously discussed^6^. After the deposition of films, no further rapid thermal anneal step was necessary.

### Magnetic hysteresis loop measurements using vibrating sample magnetometer (VSM)

Magnetic hysteresis loops of the Ce:YIG films were measured for in-plane and out-of-plane configurations at room temperature (RT). 40 magnetic moment measurements were acquired and averaged for each data point in the VSM hysteresis loops. Since the GGG substrate contribution interferes with the measurement at high field ranges (|H_applied_| > 6000 Oe) and since the films saturate below 4000 Oe, the hysteresis loops within ±4000 Oe were shown.

### Optical characterization of the films

Spectroscopic ellipsometry experiments were performed on Woollam VASE and RC2 ellipsometers at various incident angles in the photon energy range from 0.7 to 6 eV. Together with ellipsometry spectra, spectra of optical transmittance and reflectance were also recorded with the same instruments and all data were numerically treated simultaneously to ensure determination of reliable results. In the case of ellipsometry measurements carried out in reflection mode the backside of the GGG substrate was mechanically roughened to suppress spurious reflections from this interface. The measurement of depolarisation confirmed the suppression of these reflections, as the depolarisation was negligible in the entire spectral range. The sample model designed for optical spectra evaluation of Ce:YIG films consisted of a homogenous single layer on a GGG substrate. Slight surface roughness of the layers was also accounted for by the effective medium approximation. Optical properties of GGG were determined from measurements performed on uncoated substrates. The model dielectric function ε_1_ of the Ce:YIG layer was parameterized by a summation of 10 Lorentz oscillators to cover most of the spectral features presented in the experimental data.

## Additional Information

**How to cite this article**: Onbasli, M. C. *et al.* Optical and magneto-optical behavior of Cerium Yttrium Iron Garnet thin films at wavelengths of 200–1770 nm. *Sci. Rep.* **6**, 23640; doi: 10.1038/srep23640 (2016).

## Supplementary Material

Supplementary Information

Supplementary Dataset

## Figures and Tables

**Table 1:** Comparison of MO figure-of-merit values for polycrystalline Ce:YIG on non-garnet substrates, single crystalline Ce:YIG on garnet substrates (λ = 1550 nm, at RT) and bulk Ce:YIG garnets, perovskites and semiconductors.

MO Material and Substrate	MO Figure-of-merit (°dB^−1^)	Growth method	Optical Loss (dB·cm^−1^)	Reference
Y_2.82_Ce_0.18_Fe_5_O_12_ (no substrate, bulk crystal)	1420	Traveling solvent floating zone	0.52	44
Single crystalline epitaxial Ce:YIG on GGG substrates	31, 943	PLD	11.2, 6	This study
Single crystalline CeYIG on Gd_3_Sc_2_Ga_3_O_12_	340	Sputtering	9.7	23
Ce_1_Y_2_Fe_5_O_12_ on (111) doped-Gd_3_Ga_5_O_12_	321	Sputtering	14	45
Polycrystalline CeYIG on YIG deposited at 550 °C on Si substrate	20	PLD	40	25
Polycrystalline CeYIG on YIG deposited at 100 °C on Si substrate	38	PLD	29	7
Ce_1_Y_2_Fe_5_O_12_ (Ce:YIG) on Silica	56	Sputtering	48	16
Ce:YIG on Si	21.8	PLD	58	5
Fe:InP	23.8	(not mentioned)	1.66	56
Fe:InGaAsP	23	(not mentioned)	4.34	57
STCo30 (20 mTorr) on STO	0.064	PLD	390.6	58
SrTi_0.77_Co_0.23_O_3-δ_ on (001) LaAlO_3_	0.57	PLD	877	59
SrTi_0.6_Fe_0.4_O_3-δ_ on (001) LaAlO_3_	1.11	PLD	700	60

## References

[b1] Liu J., Sun X., Camacho-Aguilera R., Kimerling L. & Michel J. Ge-on-Si laser operating at room temperature. Opt. Lett. 35, 679–681 (2010). 20195317 10.1364/OL.35.000679

[b2] Onaran E. *et al.* Silicon-Germanium multi-quantum well photodetectors in the near infrared. Opt. Express 20, 7608–7615 (2012). 22453440 10.1364/OE.20.007608

[b3] Bogaerts W. *et al.* Silicon microring resonators. Laser Photon. Rev. 6, No.1, 47–73 (2012).

[b4] Reed G. T., Mashanovich G., Gardes F. Y. & Thomson D. J. Silicon optical modulators. Nature Photon. 4, 518–526 (2010).

[b5] Bi L. *et al.* On-chip optical isolation in monolithically integrated non-reciprocal optical resonators. Nature Photon. 5, 758–762 (2011).

[b6] Onbasli M. C., Goto T., Sun X., Huynh N. & Ross C. A. Integration of bulk-quality thin film magneto-optical cerium-doped yttrium iron garnet on silicon nitride photonic substrates. Opt. Express 22, 25183–25192 (2014). 25401550 10.1364/OE.22.025183

[b7] Goto T., Onbasli M. C. & Ross C. A. Magneto-optical properties of cerium substituted yttrium iron garnet films with reduced thermal budget for monolithic photonic integrated circuits. Opt. Express 20, 28507–28517 (2012). 23263087 10.1364/OE.20.028507

[b8] Stadler B. *et al.* Integration of magneto-optical garnet films by metal-organic chemical vapor deposition. IEEE Trans. Magn. 38, 1564–1567 (2002).

[b9] Gomi M., Tanida T. & Abe M. rf sputtering of highly Bi‐substituted garnet films on glass substrates for magneto‐optic memory. J. Appl. Phys. 57, 3888–3890 (1985).

[b10] Yang Q.-H., Zhang H.-W., Wen Q.-Y. & Liu Y.-L. Effects of off-stoichiometry and density on the magnetic and magneto-optical properties of yttrium iron garnet films by magnetron sputtering method. J. Appl. Phys. 108, 073901 (2010).

[b11] Yang Q., Huaiwu Z., Yingli & Qiye L. W. Effect of Post-Annealing on the Magnetic Properties of Bi:YIG Film by RF Magnetron Sputtering on Si Substrates. IEEE Trans. Magn. 43, 3652–3655 (2007).

[b12] Sung S., Qi X. & Stadler B. J. H. Integrating yttrium iron garnet onto nongarnet substrates with faster deposition rates and high reliability. Appl. Phys. Lett. 87, 121111 (2005).

[b13] Suzuki T. Magnetic and magneto‐optic properties of rapid thermally crystallized garnet films (invited). J. Appl. Phys. 69, 4756–4760 (1991).

[b14] Suzuki T., Zaharchuk G., Gorman G., Sequeda F. & Labun P. Magnetic and magneto-optical properties and crystallization kinetics of rapid-thermally crystallized Bi-substituted garnet films. IEEE Trans. Magn. 26, 1927–1929 (1990).

[b15] Vasiliev M. *et al.* Microstructural characterization of sputtered garnet materials and all-garnet magnetic heterostructures: establishing the technology for magnetic photonic crystal fabrication. J. Phys. D: Appl. Phys. 42, 135003 (2009).

[b16] Goto T. *et al.* Vacuum annealed cerium-substituted yttrium iron garnet films on non-garnet substrates for integrated optical circuits. J. Appl. Phys. 113, 17A939 (2013).

[b17] Vasiliev M. *et al.* Annealing behaviour and crystal structure of RF-sputtered Bi-substituted dysprosium iron-garnet films having excess co-sputtered Bi-oxide content. J. Phys. D: Appl. Phys. 44, 075002 (2011).

[b18] Leitenmeier S. *et al.* Studies on the growth of epitaxial bismuth-substituted iron garnet on gadolinium gallium garnet single crystals by pulsed laser deposition. J. Cryst. Growth 310, 5392–5401 (2008).

[b19] Sekhar M. C., Singh M. R., Basu S. & Pinnepalli S. Giant Faraday rotation in Bi_x_Ce_3-x_Fe_5_O_12_ epitaxial garnet films. Opt. Express 20, 9624–9639 (2012). 22535054 10.1364/OE.20.009624

[b20] Huang M. & Xu Z.-C. Liquid phase epitaxy growth of bismuth-substituted yttrium iron garnet thin films for magneto-optical applications. Thin Solid Films 450, 324–328 (2004).

[b21] Huang M. & Zhang S.-Y. Growth and characterization of cerium-substituted yttrium iron garnet single crystals for magneto-optical applications. Appl. Phys. A 74, 177–180 (2002).

[b22] Mino S., Tate A., Uno T., Shintaku T. & Shibukawa A. Properties of Ce-Substituted Yttrium Iron Garnet Film Containing Indium Prepared by RF-Sputtering. Jpn. J. Appl. Phys. 32, L994–L996 (1993).

[b23] Shintaku T., Tate A. & Mino S. Ce-substituted yttrium iron garnet films prepared on Gd_3_Sc_2_Ga_3_O_12_ garnet substrates by sputter epitaxy. Appl. Phys. Lett. 71, 1640–1642 (1997).

[b24] Bi L., Hu J., Kimerling L. & Ross C. A. Fabrication and characterization of As_2_S_3_/Y_3_Fe_5_O_12_ and Y_3_Fe_5_O_12_/SOI strip-loaded waveguides for integrated optical isolator applications. Proc. SPIE 7604, 760406 (2010).

[b25] Bi L., Hu J., Dionne G. F., Kimerling L. & Ross C. A. Monolithic integration of chalcogenide glass/iron garnet waveguides and resonators for on-chip nonreciprocal photonic devices. Proc. SPIE 7941, 794105 (2011).

[b26] Mizumoto T., Takei R. & Shoji Y. Waveguide Optical Isolators for Integrated Optics. IEEE J. Quant. Electron. 48, 252–260 (2012).

[b27] Tien M.-C., Mizumoto T., Pintus P., Kromer H. & Bowers J. E. Silicon ring isolators with bonded nonreciprocal magneto-optic garnets. Opt. Express 19, 11740–11745 (2011). 21716405 10.1364/OE.19.011740

[b28] Goto T. *et al.* A nonreciprocal racetrack resonator based on vacuum-annealed magnetooptical cerium-substituted yttrium iron garnet. Opt. Express 22, 19047–19054 (2014). 25320991 10.1364/OE.22.019047

[b29] Levy M. The on-chip integration of magnetooptic waveguide isolators. IEEE J. Sel. Top. Quantum Electron. 8, 1300–1306 (2002).

[b30] Dai D., Bauters J. & Bowers J. E. Passive technologies for future large-scale photonic integrated circuits on silicon: polarization handling, light non-reciprocity and loss reduction. Light Sci. Appl. 1, e1 (2012).

[b31] Sepulveda B., Armelles G. & Lechuga L. M. Magneto-optical phase modulation in integrated Mach–Zehnder interferometric sensors. Sensor Actuat A-Phys. 134, 339–347 (2007).

[b32] Bi L. *et al.* Magneto-Optical Thin Films for On-Chip Monolithic Integration of Non-Reciprocal Photonic Devices. Materials 6, 5094–5117 (2013). 10.3390/ma6115094 PMC5452783 28788379

[b33] Kehlberger A. *et al.* Enhanced Magneto-optic Kerr Effect and Magnetic Properties of CeY_2_Fe_5_O_12_ Epitaxial Thin Films. Phys. Rev. Appl. 4, 014008 (2015).

[b34] Gomi M., Furuyama H. & Abe M. Strong magneto-optical enhancement in highly Ce-substituted iron garnet films prepared by sputtering. J. Appl. Phys. 70, 7065–7067 (1991).

[b35] Visnovsky S. In Optics in Magnetic Multilayers and Nanostructures (CRC Taylor & Francis, 2006).

[b36] Lang M. *et al.* Proximity Induced High-Temperature Magnetic Order in Topological Insulator - Ferrimagnetic Insulator Heterostructure. Nano Lett. 14, 3459–3465 (2014). 24844837 10.1021/nl500973k

[b37] Jiang B. *et al.* Spectral properties and charge transfer luminescence of Yb^3+^:Gd_3_Ga_5_O_12_ (Yb:GGG) crystal. J. Cryst. Growth 277, 186–191 (2005).

[b38] Wettling W., Andlauer B., Koidl P., Schneider J. & Tolksdorf W. Optical absorption and Faraday rotation in yttrium iron garnet. Phys. Status Solidi B 59, 63–70 (1973).

[b39] Visnovsky S., Thuy N. P., Stepanek J., Prosser V. & Krishnan R. Magnetooptical spectra of Y_3_Fe_5_O_12_ and Li_0.5_Fe_2.5_O_4_ between 2.0 and 5.8 eV. J. Appl. Phys. 50, 7466–7468 (1979).

[b40] Kahn F. J., Pershan P. S. & Remeika J. P. Ultraviolet Magneto-Optical Properties of Single-Crystal Orthoferrites, Garnets, and Other Ferric Oxide Compounds. Phys. Rev. 186, 891–918 (1969).

[b41] Wemple S. H., Blank S. L., Seman J. A. & Biolsi W. A. Optical properties of epitaxial iron garnet thin films. Phys. Rev. B 9, 2134–2144 (1974).

[b42] Jakubisova-Liskova E., Visnovsky S., Chang H. & Wu M. Optical spectroscopy of sputtered nanometer-thick yttrium iron garnet films. J. Appl. Phys. 117, 17B702 (2015).

[b43] Diercks G. J. Jr. & Samuelson S. Magneto-optical properties of Y_3-x-y_Ce_x_La_y_Fe_5_O_12_. IEEE Trans. Magn. 31, 3328–3330 (1995).

[b44] Higuchi S., Furukawa Y., Takekawa S., Kamada O. & Kitamura K. Magneto-Optical Properties of Cerium-Substituted Yttrium Iron Garnet Single Crystals Grown by Traveling Solvent Floating Zone Method. Jpn. J. Appl. Phys. 38, 4122–4126 (1999).

[b45] Shintaku T. Integrated optical isolator based on efficient nonreciprocal radiation mode conversion. Appl. Phys. Lett. 73, 1946–1948 (1998).

[b46] Gomi M., Furuyama H. & Abe M. Enhancement of Faraday Effect in Highly Ce-Substituted YIG Epitaxial Films by RF Sputtering. Jpn. J. Appl. Phys. 29, L99–L100 (1990).

[b47] Gomi M., Satoh K. & Abe M. Giant Faraday Rotation of Ce-Substituted YIG Films Epitaxially Grown by RF Sputtering. Jpn. J. Appl. Phys. 27, L1536–L1538 (1988).

[b48] Gomi M., Furuyama H. & Abe M. Strong magneto‐optical enhancement in highly Ce‐substituted iron garnet films prepared by sputtering. J. Appl. Phys. 70, 7065–7067 (1991).

[b49] Kucera M., Bok J. & Nitsch K. Faraday rotation and MCD in Ce doped yig. Solid State Commun. 69, 1117–1121 (1989).

[b50] Xu Y., Yang J. H. & Zhang G. A theoretical investigation on the strong magneto-optical enhancement in Ce-substituted yttrium iron garnet. J. Phys. Condens. Matter 5, 8927–8934 (1993). 10.1103/physrevb.50.13428 9975535

[b51] Wittekoek S., Popma T. J. A., Robertson J. M. & Bongers P. F. Magneto-optic spectra and the dielectric tensor elements of bismuth-substituted iron garnets at photon energies between 2.2–5.2 eV. Phys. Rev. B 12, 2777–2788 (1975).

[b52] Veis M. *et al.* Polar and longitudinal magneto-optical spectroscopy of bismuth substituted yttrium iron garnet films grown by pulsed laser deposition. Thin Solid Films 519, 8041–8046 (2011).

[b53] Kim T. & Shima M. Reduced magnetization in magnetic oxide nanoparticles. J. Appl. Phys. 101, 09M516 (2007).

[b54] Fujiwara H. In Spectroscopic ellipsometry: Principles and applications (John Wiley & Sons, 2007).

[b55] Remes Z., Vasudevan R., Jarolimek K., Smets A. H. M. & Zeman M. The optical spectra of a-Si:H and a-SiC:H thin films measured by the absolute photothermal deflection spectroscopy (PDS)). Solid State Phenom. 213, 19–28 (2014).

[b56] Zaman T. R., Guo X. & Ram R. J. Faraday Rotation in Semiconductors For Photonic Integration. Proc. Conf. Lasers Electro-Optics p. 2 (OSA, San Francisco, 2004).

[b57] Zaman T. R., Guo X. & Ram R. Faraday rotation in an InP waveguide. J. Appl. Phys. 90, 023514 (2007).

[b58] Onbasli M. C. *et al.* Oxygen partial pressure dependence of magnetic, optical and magneto-optical properties of epitaxial cobalt-substituted SrTiO_3_ films. Opt. Express 23, 13399–13409 (2015). 26074589 10.1364/OE.23.013399

[b59] Bi L., Kim H. S., Dionne G. F. & Ross C. A. Structure, magnetic properties and magnetoelastic anisotropy in epitaxial Sr(Ti_1−x_Co_x_)O_3_ films. New J. Phys. 12, 043044 (2010).

[b60] Kim H.-S., Bi L., Dionne G. F. & Ross C. A. Magnetic and magneto-optical properties of Fe-doped SrTiO_3_ films. Appl. Phys. Lett. 93, 092506 (2008).

