# Supplementary material for: Optical and magneto-optical behavior of Cerium Yttrium Iron Garnet thin films at wavelengths of 200–1770 nm
Source: Sci Rep. 2016 Mar 30;6:23640. doi: 10.1038/srep23640 (PMC4812311; doi:10.1038/srep23640)
Supplement: Supplementary Information [file srep23640-s1.pdf]

Supplementary Information for “Optical and magneto-optical behavior of Cerium Yttrium Iron Garnet thin films at wavelengths of 200 - 1770 nm”

Mehmet C. Onbasli[1,a)], Lukáš Beran[2,a)], Martin Zahradník[2], Miroslav Kučera[2], Roman Antoš[2], Jan Mistrík[3], Gerald F. Dionne[1], Martin Veis[2,**] and Caroline A. Ross[1,*]

[1] Department of Materials Science and Engineering, Massachusetts Institute of Technology, 77 Massachusetts Avenue, MIT Cambridge, MA 02139, USA
[2] Charles University of Prague, Faculty of Mathematics and Physics, KeKarlovu 3, 12116 Prague 2, Czech Republic
[3] University of Pardubice, Faculty of Chemical Technology, Institute of Applied Physics and Mathematics, Studentska 95, 53210 Pardubice, Czech Republic

[a)]equal contribution
Correspondence and requests for materials should be addressed to:
*caross@mit.edu, **veis@karlov.mff.cuni.cz

Keywords: Magneto-optical materials; Isolators; Pulsed laser deposition; Iron garnets; nonreciprocal

In this supplementary information section, we present x-ray diffraction (ω-2θ) scans of the films and the substrates in Fig. S1 in order to demonstrate that the samples have no secondary phases. In the second figure, Fig. S2, the substrate orientation and angular dependence of the magnetic anisotropy properties extracted from the in-plane (IP) magnetic hysteresis loop measurements are presented.

The crystalline phase content of the films was characterized using XRD (ω–2θ) scans and a silicon zero-background holder. PANalytical X'Pert Pro Multipurpose Diffractometer with Bragg-Brentano Optics (λ=1.5406 Å Cu K_α) was used for the ω–2θ scans. The substrates were not tilted during measurement so the substrate peaks are visible. The ω-2θ plots in Fig. S1(a) were measured for Ce:YIG/GGG (100) and a GGG (100) substrate with no film. The plot shows the (400) peak originating from the film while the rest of the peaks are from the substrate. Fig. S1(b, c) show corresponding data from films on (110) and (111) substrates respectively. The vertical offset in Fig. S1(c) originates from the intensity drift of the emitted x-ray power from the Copper x-ray source and is not related to the samples. The substrate peaks for $K\alpha_1$ and $K\alpha_2$ x-ray signals show up as splittings near $2\theta = 29°$ for Fig. S1a, as splittings near $2\theta = 20.3°$ for Fig. S1b, and as splittings near $2\theta = 51°$ in Fig. S1c.

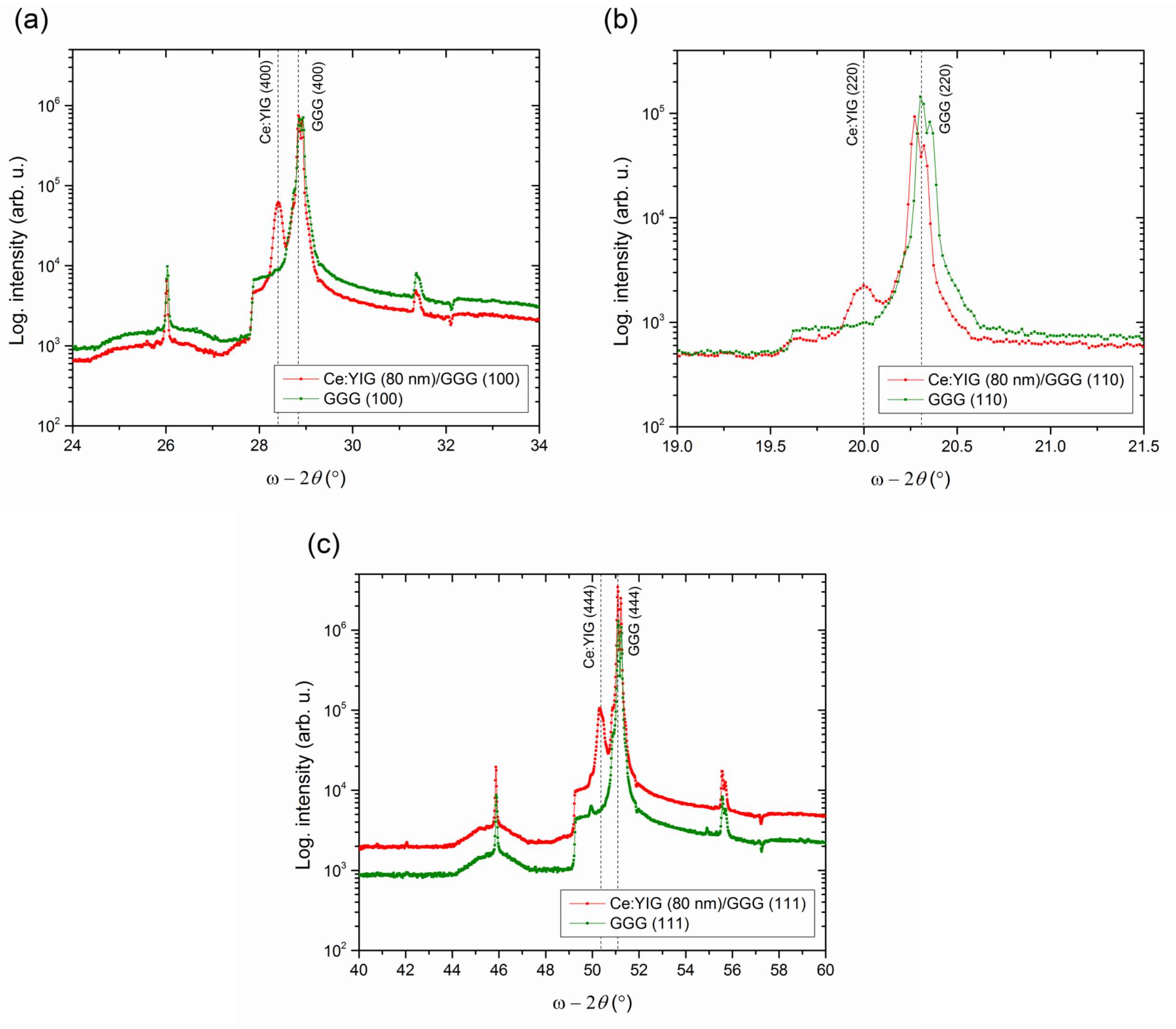

Figure S1. XRD patterns of Ce:YIG (80 nm) films on (a) GGG (100), (b) GGG (110), and (c) GGG (111) substrates. Y_2O_3 or CeO_2 peaks were not found. Each plot contains an XRD pattern for a similar substrate without film. All the films are single phase garnet. Major garnet peaks are indicated with dashed lines.

IP room-temperature magnetic hysteresis loops were measured for each film for every 10° direction in plane. The substrate contributions were subtracted for each loop. Next, the angular dependence of H_c (coercivity) and M_r/M_s were recorded and presented in polar plots. The angular dependence of IP coercivity (H_c) for Ce:YIG on GGG (100), (110), and (111) are given in Fig. S2 (a, c, e), respectively.

Angular dependence of in-plane squareness (defined as magnetic remanence/saturation; M_r/M_s) for Ce:YIG (80 nm) on GGG (100), (110), and (111) are in Fig. S2 (b, d, f), respectively. Ce:YIG on GGG (100) exhibits 4-fold cubic symmetry and the IP <011> are the magnetic easy directions, which appear along 45, 135, 225 and 315° IP in Figures 3(a, b). This agrees with a previous study of Ce:YIG/GGG (100) [1]. Ce:YIG on GGG (110) exhibits a clear 2-fold symmetry. Ce:YIG on GGG (111) would be expected to show a 6-fold symmetry as seen in [1], but the modulation in properties is small. These measurements are consistent with angularly dependent magneto-optical measurements of the longitudinal Kerr effect at remanence (not shown here).

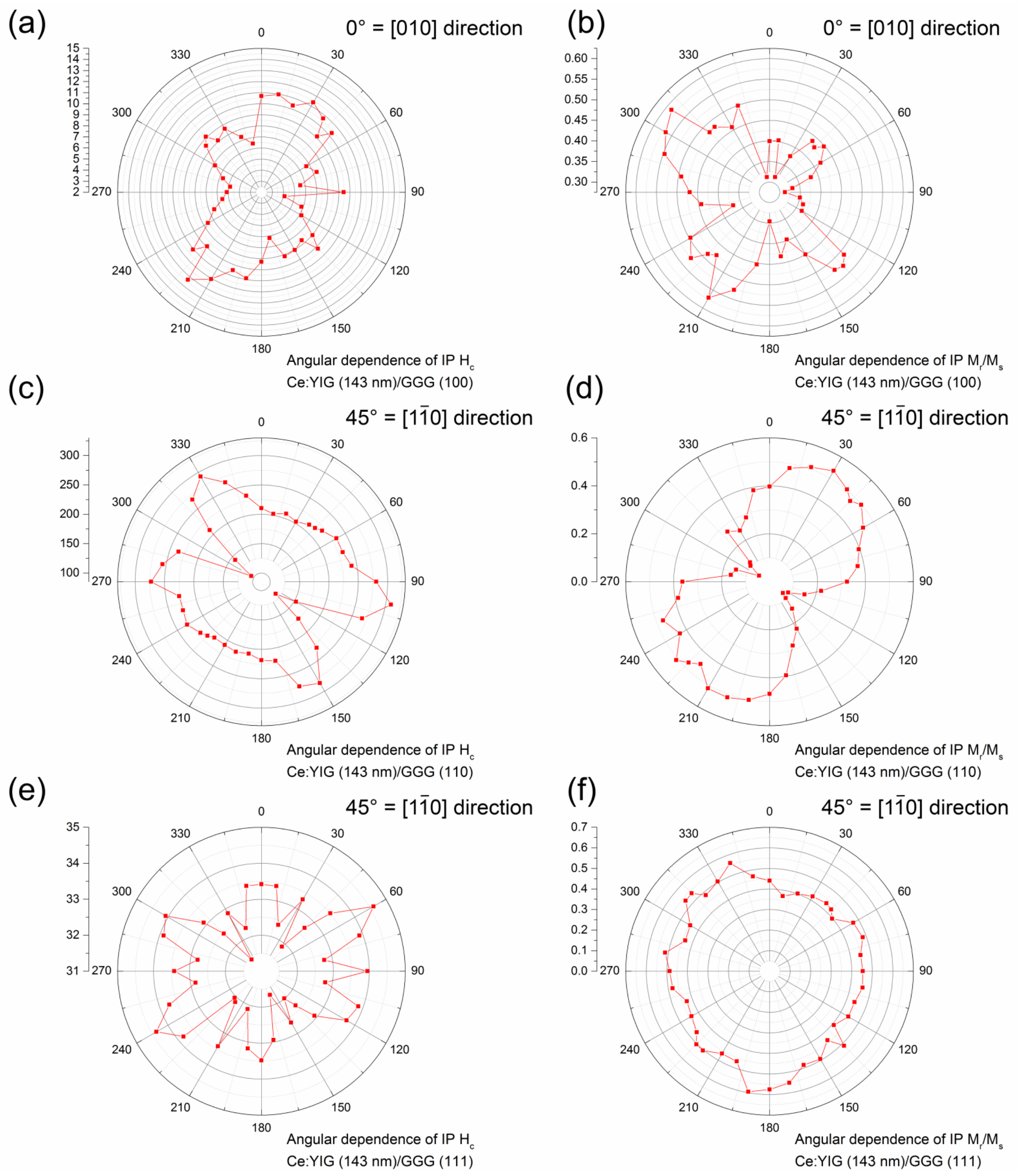

Figure S2. (a, c, e) Angular dependence of in-plane coercivity (H_c, with the scale in Oe) for Ce:YIG (80 nm) on GGG (100), (110), and (111), respectively. (b, d, f) Angular dependence of in-plane squareness (defined as remanence/saturation; M_r/M_s) for Ce:YIG (80 nm) on GGG (100), (110), and (111), respectively. The film on GGG (100) shows 4-fold symmetry, the film on GGG (110) shows 2-fold symmetry, and the film on GGG (111) shows weak 6-fold symmetry.

References

1. Kehlberger, A., Richter, K., Jakob, G., Onbasli, M. C., Kim, D. H., Goto, T., Ross, C. A., Gotz, G., Reiss, G., Kuschel, T., Klaui, M. Enhanced Magneto-optic Kerr Effect and Magnetic Properties of $CeY_2Fe_5O_{12}$ Epitaxial Thin Films *Phys. Rev. Applied* **4,** 014008 (2015).
